# Comparison of Diagnostic Modalities for Carpal Tunnel Syndrome: A Systematic Review

**DOI:** 10.7759/cureus.92577

**Published:** 2025-09-17

**Authors:** Mikahla E Gay, Jose G Lima, Vanessa Vieites, Starlie C Belnap, Richard Morgan

**Affiliations:** 1 Medicine, Florida International University, Herbert Wertheim College of Medicine, Miami, USA; 2 Neuroscience, Baptist Health South Florida, Miami, USA; 3 Physical Medicine &amp; Rehabilitation, Baptist Health South Florida, Miami, USA

**Keywords:** a systematic review, carpal tunnel syndrome, diagnostic accuracy, diagnostic accuracy analysis, diagnostic ultrasound of peripheral nerves, electromyography (emg), nerve conduction studies (ncs), physical medicine and rehabilitation, point-of-care ultrasound (pocus), ultrasonography (usg)

## Abstract

Carpal tunnel syndrome (CTS) is a common nerve entrapment disorder that affects millions, particularly older adults and women. It causes numbness, tingling, and discomfort in the hand, primarily affecting the median nerve. Risk factors include occupation (e.g., jobs involving repetitive motion or vibrating tools) and conditions such as obesity, pregnancy, and diabetes. Accurate diagnosis is critical for effective treatment, with nerve conduction studies (NCS) and ultrasound (US) being two key diagnostic methods. Each approach has strengths, limitations, and diagnostic utility, necessitating a comparison to determine the best use case for each tool. This comprehensive systematic review aims to compare the diagnostic accuracy and clinical utility of NCS and US in diagnosing CTS and provide conclusions on the most appropriate modality in different clinical scenarios.

A PubMed literature review conducted in August 2024 identified 49 studies. Of these, 31 were excluded for design and non-English language, evaluating only a single modality, or lacking comparison of diagnostic utility. The remaining 18 studies, published between 2008 and 2023, directly comparing NCS and US for diagnosing CTS were included. We included studies published within 20 years that directly compared measures of diagnostic accuracy, disease severity, and identification of anatomic abnormalities between US and NCS. Novel approaches, such as the inching technique, and newer measurements, such as transverse carpal ligament thickness, were also considered. Descriptive statistics were calculated, and Welch’s t-test was used to compare means, with p-values indicating statistical significance.

A total of 18 studies met the inclusion criteria for analysis and included cohort (n=9), case-control (n=7), cross-sectional (n=1), and case-series (n=1) designs. NCS demonstrated high diagnostic accuracy, with an average sensitivity of 83% and improved sensitivity up to 96.7% using the inching technique. The comparison showed a higher average sensitivity for US than NCS (84.9% vs. 83.3%, respectively). Similarly, US had a higher average specificity than NCS (71.2% vs. 66.6%, respectively). The differences between the sensitivity and specificity of US and NCS were not statistically significant (p=0.76 and p=0.72, respectively). Studies comparing NCS and US showed that each method offers distinct advantages based on clinical severity, with US being more sensitive for structural assessment and NCS offering better functional insights.

Both NCS and US have unique diagnostic roles in CTS. While NCS remains the gold standard for evaluating nerve function, US offers a viable, less invasive alternative, particularly for detecting early structural changes. Patient-specific factors, clinical presentation, and disease severity should guide the choice between the two. This study, however, is limited by its design, the small number of eligible high-quality studies, and conflicting evidence among them. While the review employed robust methodology, its narrow focus restricted the pool of available research. Variability in study populations, methodologies, and outcome measures further contributed to inconsistent findings, underscoring the need for high-quality, standardized studies. Further research is needed to refine diagnostic protocols, especially when using both techniques to enhance diagnostic accuracy and patient outcomes.

## Introduction and background

Carpal tunnel syndrome (CTS) is a nerve entrapment pathology where the median nerve is compressed as it passes through the carpal tunnel. This pathology affects millions of people around the world. The condition is characterized by reportedly painful or uncomfortable numbness and tingling within the median nerve distribution of the fingers and inner palm. CTS is the most common form of median nerve compression reported [1], especially in women and elderly patients. As patients age, their risk of developing CTS increases, and women have a higher incidence of developing the condition than men, with an annual incidence rate of 9.2% in women and 6% in men [2]. Furthermore, women between the ages of 45-54 years and men between the ages of 75-84 years have an increased incidence of developing CTS than their younger counterparts [3]. 

Studies suggest that many cases of CTS are occupation-related; for example, patients who work jobs involving vibrating objects, typing, or fishing have an increased risk of developing CTS [4]. However, CTS can also be idiopathic, arising spontaneously from an unknown cause [1]. There are many well-documented risk factors for CTS, including pregnancy, obesity, menopause, kidney failure, hypothyroidism, diabetes mellitus, congestive heart failure, and rheumatoid inflammation [5]. Given the numerous factors that contribute to the development of CTS, accurate diagnosis is essential. 

CTS can be diagnosed clinically and through diagnostic studies, including nerve conduction studies (NCS) or ultrasound (US). Accurate diagnosis requires consideration of the advantages and disadvantages of each method. Diagnostic accuracy depends on the sensitivity and specificity of various methods, where sensitivity is defined as the proportion of positive results among patients with the disease and specificity is defined as the proportion of negative results among patients who do not have the disease. CTS diagnosis involves a physical examination with specific maneuvers to reproduce symptoms. One such test is Tinel’s sign, which involves tapping the carpal tunnel to elicit the characteristic pins and needles sensation in the patient’s fingers. A positive result indicates CTS with 59% sensitivity [6]. Another option is Durkan’s test, which involves pressing on the carpal tunnel for 30 seconds, and a positive result has 67% sensitivity [6]. The most sensitive maneuver, Phalen’s Test, requires patients to flex their wrists together for 60 seconds, with symptom reproduction indicating CTS in 70% of cases [6]. Other methods of diagnosing CTS include self-report surveys, such as the CTS-6, Boston Carpal Tunnel Questionnaire (BCTQ), and historical-objective-distribution-based (Hi-Ob-Db) scale [7]. Clinical diagnosis is fast, low-cost, and well-tolerated but less sensitive than electrodiagnostic tests and imaging, emphasizing the importance of more accurate complementary tools. 

Diagnosing CTS**

NCS is one of the most reliable tools used to accurately diagnose CTS. NCS measures the electrical activity of the nerve, namely velocity and amplitude, while offering physicians insights into the health of the nerve. Slower velocity suggests nerve compression, while reduced amplitude indicates nerve damage. NCS has an 88% accuracy rate, meaning it can detect correctly CTS in 88% of cases [8]. Inching techniques, which stimulate the median nerve at 1 cm intervals, improve accuracy by pinpointing damaged areas, showing 92.5% sensitivity in recent studies [9]. Despite their accuracy, NCS and inching techniques have drawbacks, such as cost, time, and patient discomfort. 

US is another tool physicians use to confirm a diagnosis of CTS through measurement of the median nerve cross-sectional area at the level of the inlet. Studies have found the highest sensitivity is achieved by measuring the cross-sectional area at the pisiform level [10]. Some studies suggest using US as a first-line diagnostic option in patients with an 80% pre-test probability, meaning there is an 80% chance that a patient has CTS before diagnostic testing [10]. A recent systematic review found US sensitivity ranges from 63% to 96.9% [11]. Despite variability in sensitivity, US is a widely supported diagnostic modality due to its affordability and efficiency; however, results vary depending on the operator’s skill level and the chosen cross-sectional area cutoff for diagnosis [12]. 

The current study 

Current guidelines from American Academy of Orthopaedic Surgeons (AAOS) state that CTS diagnosis should be established using the CTS-6. The guidelines also support using US or NCS when their positive predictive value is low. AAOS does not establish a preference for using US vs. NCS, stating that there is no significant difference between these diagnostic tests [13]. Although NCS has long been considered the gold standard, recent studies offer mixed evidence for both methods. This literature review aims to compare the diagnostic utility of US and NCS in accurately diagnosing CTS and to evaluate how each modality correlates with the clinical presentation. 

This article was previously presented as a meeting abstract at the 2024 American Medical Association Poster Showcase on November 8, 2024. 

Methods 

**Figure 1:**
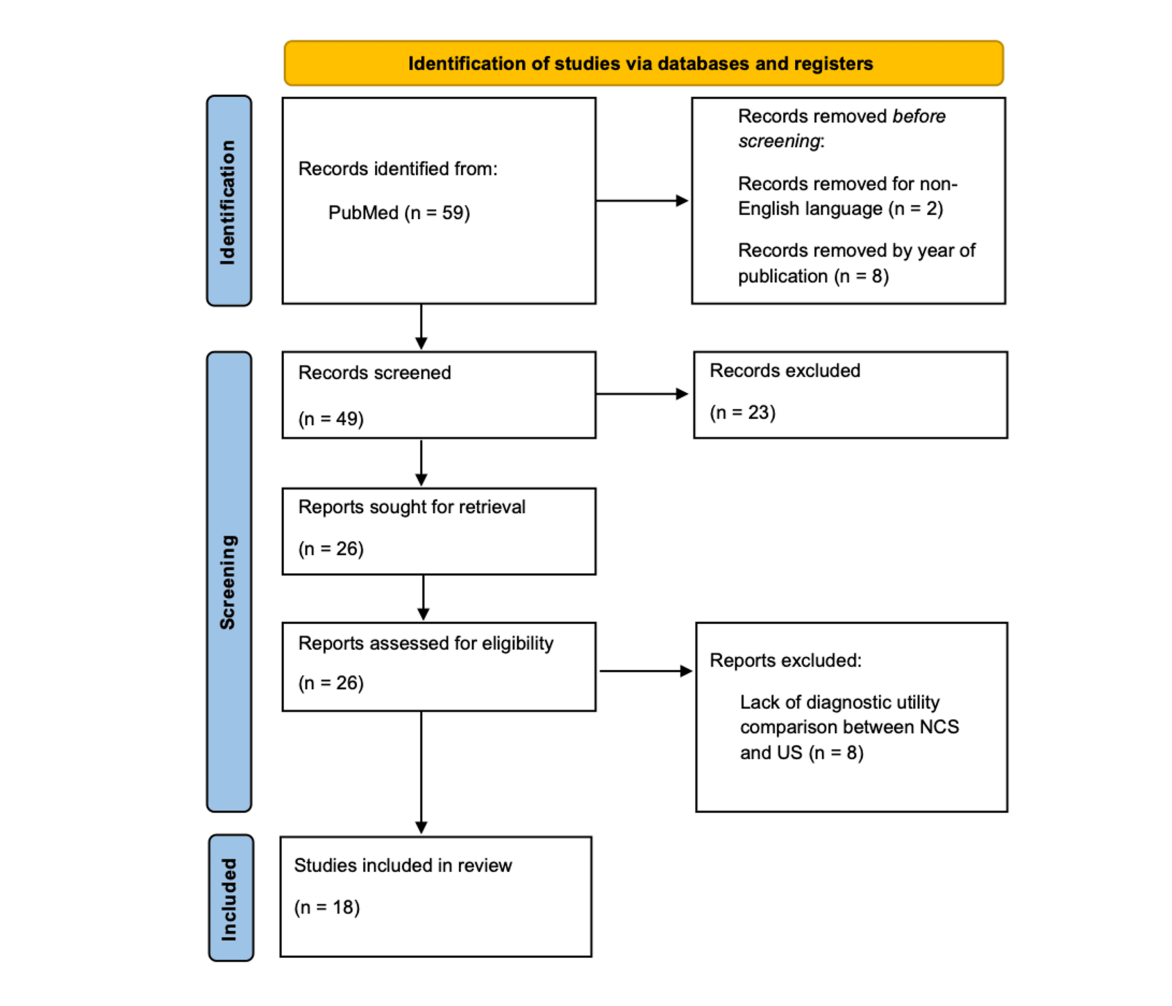
PRISMA diagram for identification and selection of studies PRISMA, Preferred Reporting Items for Systematic reviews and Meta-Analyses

A PubMed search was conducted in August 2024 using the medical subject headings (MeSH) terms “electromyography”, “ultrasonography”, and “carpal tunnel syndrome.” These MeSH terms were chosen to compare electromyography (EMG) or NCS and ultrasonography for diagnosis of CTS. Additional filters to exclude studies published greater than 20 years ago and in non-English language were applied. Given recent advancements in US imaging and technology, we chose to analyze studies from 2004 to 2024 to capture the variation in diagnostic accuracy over time. The initial search using these search terms yielded 59 studies, and an additional 10 were removed with filters for English and publication date between 2004 and 2024. Articles were included for review that directly compared measures of diagnostic accuracy, disease severity, or identification of anatomic abnormalities between US and NCS. Exclusion criteria included articles published in any language other than English, case reports, and studies that only examined one diagnostic modality. The title and full-text reviews were conducted by two independent researchers and included studies were agreed upon after individual review. We excluded 23 studies based on title and abstract review due to case report design (n=7) and failure to discuss both US and NCS (n=16). Upon full-text review, an additional eight studies were excluded for lack of diagnostic utility comparison. Figure 1 details the identification and selection of studies.

For each study, we collected data on study population size, measurements and cutoff values for diagnosis of CTS through US and NCS, clinical reference tools, disease severity, and diagnostic accuracy through measures of sensitivity and specificity. Studies reporting sensitivity and specificity were grouped for analysis of diagnostic accuracy. Descriptive statistics, including means for continuous variables, were calculated to summarize the data. Welch’s t-test was performed to compare means, and p-values were used to determine statistical significance. 

## Review

The systematic review included 18 studies conducted between 2008 and 2023. Nine studies used a cohort design, while seven implemented a case-control design to compare the US and NCS for diagnosing and characterizing CTS. The remaining two studies were cross-sectional and case-series designs. Two of the 18 studies analyzed NCS with an inching technique. Table 1 provides a summary of the study characteristics in this review.

**Table 1:** Summary of study characteristics in systematic review AANEM, American Association of Neuromuscular & Electrodiagnostic Medicine; BCTQ, Boston Carpal Tunnel Questionnaire; CMAP, Compound muscle action potential; CSA, Cross-sectional area; CTS, Carpal tunnel syndrome; DML, Distal motor latency; DSAP, Distal sensory action potential; DSL, Distal sensory latency; EDx, Electrodiagnostic (3 vs. 5, refer to the number of classifications in the scale); EMG, Electromyography; Hi-Ob, Historical-objective scale; Hi-Ob-Db, Historical-objective-distribution based scale; HRUS, High-resolution ultrasonography; MCV, Motor conduction velocity; m-CSA, Median nerve cross-sectional area; m-CSA-I, Median nerve cross-sectional area at inlet (before flexor retinaculum); m-CSA-M, Median nerve cross-sectional area at middle (level of scaphoid tubercle); m-CSA-O, Median nerve cross-sectional area at outlet (at hook of hamate); NCS, Nerve conduction studies; SCV, Sensory conduction velocity; SML, Sensory motor latency; SNAP, Sensory nerve action potential; TCL, Transverse carpal ligament (at trapezius hook of hamate ligament); u-CSA, Ulnar nerve cross-sectional area; VAS, Visual analog score; WFR, Wrist-forearm ratio

Author (year) (citation)	Study design	Size	Cases	NCS measurements	US measurements	Clinical reference	NCS severity scale
Ari (2022) [14]	Cohort	58 patients	Positive NCS, moderate or severe	DSAP, SCV, difference median-ulnar DSAP	m-CSA-I, TCL thickness	BCTQ, VAS	Stevens scale
Aseem (2017) [15]	Cohort	14 patients/22 hands	Clinical CTS, normal NCS	SCV, sensory amplitude, DML, motor amplitude	m-CSA at maximum enlargement, WFR	Symptoms only	N/A
Azadeh (2017) [16]	Case control	32 hands, 30 controls	Clinical CTS, positive NCS	Sensory antidromic peak latency, DML at 8 points	AP and transverse cross-sectional diameter at 8 points (4 cm distal and 3 cm proximal to the distal wrist crease)	Symptoms only	-
Chen (2023) [17]	Cohort	265 patients/402 hands	Clinical CTS with available CTS-6, NCS, and US	DSL, CMAP, SNAP, median-ulnar relative latency	m-CSA-I	CTS-6	-
Chompoopong (2021) [18]	Cohort	415 patients	Positive NCS or positive HRUS	MCV, DML, CMAP, SCV, SNAP (AANEM)	m-CSA, WFR	Symptoms only	Stevens scale
Drakopoulos (2019) [19]	Cohort	96 patients	Clinical CTS	-	m-CSA-I, proximal carpal tunnel	Symptoms only	Padua scale
Fowler (2014) [20]	Cohort	85 patients	Clinical CTS referred for EMG	DML, DSL	m-CSA-I	CTS-6	N/A
Kanikannan (2015) [21]	Case control	57 patients/92 hands, 50 controls/100 hands	Clinical CTS	DML, DSL	m-CSA-I	Symptoms only	Bland scale
Kwon (2008) [22]	Case control	29 patients/41 hands, 29 controls/41 wrists	Clinical CTS	SNAP, DSL, DML, CMAP	m-CSA-I, m-CSA-O	BCTQ	-
Mhoon (2012) [23]	Case control	100 patients/192 wrists, 25 controls/50 wrists	Clinical CTS referred for EMG	DML, mixed nerve latency, median-ulnar mixed latency	m-CSA-I, WFR	Hi-Ob-Db	EDX 3, EDX 5
Paliwal (2014) [24]	Cohort	77 patients/127 hands, 35 controls	Positive NCS	DSL, DML, lumbrical interossei latency difference	m-CSA-I, m-CSA-O, forearm m-CSA	-	Stevens scale
Salman (2018) [25]	Case control	203 patients, 113 controls	Positive NCS	MCV, DML, CMAP, SCV, SNAP (AANEM)	m-CSA at the precanal, inlet, midcanal, outlet, antecubital, inlet/antecubital CSA ratio	Symptoms only	Stevens scale
Shields (2021) [26]	Case series	25 patients/30 hands	Acute CTS	DML, SML, CMAP, SNAP	m-CSA-I	Symptoms only	-
Singla (2020) [27]	Case control	31 patient/56 hands, 25 controls/50 hands	Clinical CTS	MCV, DML, CMAP, SCV, SNAP (AANEM)	m-CSA-I, m-CSA-O, flattening ratio, AP diameter of the carpal tunnel	Hi-Ob	Padua scale
Stonsaovapak (2022) [28]	Cross-sectional	40 hands, 15 control hands	Positive NCS	DSL, DML, SNAP, CMAP, SCV, MCV at 11 points (6 cm distal and 4 cm proximal to distal wrist crease)	m-CSA at 11 points (6 cm distal and 4 cm proximal to distal wrist crease)	Symptoms only	AANEM
Atan (2018) [29]	Case control	50 patients, 50 controls	Positive NCS	-	m-CSA-I, u-CSA, m-CSA/u-CSA	BCTQ	Padua scale
Mondelli (2008) [30]	Cohort	85 patients	Clinical CTS, mild symptoms	MCV, DML, CMAP, SCV, SNAP (AANEM)	m-CSA-I, m-CSA-O, m-CSA-M	Hi-Ob, BCTQ	Padua scale
Visser (2008) [31]	Cohort	168 patients, 137 controls	Clinical CTS	MCV, DML, CMAP, SCV, SNAP (AANEM)	m-CSA-I, distal one-third level of the forearm	Symptoms only	Padua scale

Measurements

The most common measurements for NCS included median nerve sensory latency, median nerve motor latency, median conduction velocity, compound muscle action potential (CMAP), and sensory nerve action potential (SNAP). Diagnostic criteria varied minimally between individual practice guidelines. Cutoff ranges for diagnosis of CTS were as follows: median nerve sensory latency greater than 3.2-3.7 ms, median nerve motor latency greater than 4.0-4.3 ms, median conduction velocity less than 49-50 m/s, CMAP less than 10 mV, and SNAP greater than 6 µV.

Routine ultrasonographic measurements included median nerve cross-sectional area (m-CSA) and wrist-forearm ratio (WFR). Like NCS, these measurements varied minimally across practices, with m-CSA ranges greater than 9-13 mm^2^ and WFR greater than 1.4-1.5 establishing the diagnosis. Other measurements, such as the flattening ratio, were included, but only clinically significant measurements were analyzed.

While most studies used a standard cutoff as defined by previous literature [14-28], other studies conducted receiver operating curve analyses or control comparisons to determine the cutoff within their specific patient populations [29-31]. Additionally, studies differed in the methodology of measurements, such that some measured the CSA once, while others collected 3-5 CSA measurements and averaged the values.

Novel measurements

Some studies explored the diagnostic utility of novel measurements, including transverse carpal ligament (TCL) thickness [14], the ratio of m-CSA to ulnar nerve cross-sectional area (u-CSA) [29], and m-CSA at the carpal tunnel outlet (CTO) [24]. Clinical or diagnostic utility has been demonstrated for both TCL thickness and CTO m-CSA. However, the ratio of m-CSA to u-CSA displayed similar sensitivity and specificity in detecting EMG-confirmed CTS compared to m-CSA alone. Specifically, the sensitivity was 86% for m-CSA/u-CSA and 80% for m-CSA, while the specificity was 72% for m-CSA/u-CSA and 80% for m-CSA [29]. TCL thickness was not linked to symptom duration or EMG findings but was strongly correlated with symptom severity as measured by the BCTQ [14].

Inching technique

Two studies compared sonography and NCS with an inching technique. Azadeh and colleagues used the 8-point inching technique, measuring from 3 cm proximal to 4 cm distal to the distal wrist, while Stonsaovapak and colleagues used an 11-point inching technique, measuring from 4 cm proximal to 6 cm distal to the distal wrist crease [16,28]. Using the 11-point technique, researchers found that measuring the same points using US produced reliable measurements from 4 cm proximal to 4 cm distal to the distal wrist crease due to bifurcation of the median nerve [16,28].

Regarding NCS, both studies found that segments 2 cm and 3 cm distal to the distal wrist crease produced statistically significant antidromic latency differences compared to other segments. Antidromic peak latency analysis revealed that the 3 cm distal segment had 71.9% sensitivity, and the 1 cm distal segment had 96.6% specificity [16]. Motor nerve analysis, however, showed that the 3 cm distal segment had 96.7% sensitivity, and the 2 cm distal segments had 65.2% specificity. Both US studies found that mean m-CSAs were significantly greater in nearly all measured points in patients with CTS compared with controls [16,28]. Stonsaovapak and colleagues found these differences were insignificant at the segments 3 cm and 4 cm proximal to the distal wrist crease [28]. This study also reported slight, measurable differences between mild and moderate cases of CTS on US. In moderate cases, m-CSAs were greater at the distal wrist crease, 1 cm and 3 cm distally [28].

Correlations between NCS and US

Of the 16 experimental design studies included in the review, seven studies defined cases of CTS using NCS, excluding patients with a normal NCS [14,16,17,24-26,28]. Eleven studies used NCS to determine the severity of CTS, but only five of these studies used a validated tool as a reference to analyze clinical severity. Among the various evidence-based NCS severity scales implemented, scales validated by Padua and colleagues [32] (n=5) and Stevens and colleagues [33] (n=4) were most cited. Other NCS severity scales included those proposed by the American Association of Neuromuscular & Electrodiagnostic Medicine (AANEM) guidelines, Bland and colleagues [34], and individual laboratory guidelines (e.g., EDX 3 scale designed by Duke University).

There were statistically significant differences between US findings of positive and negative NCS cases. Five studies showed a significant relationship between CSA measurements and NCS severity [19,23,25,27,31], while one study showed no significant difference between moderate and severe cases [14]. Mondelli and colleagues measured the CSA at the inlet, outlet, and middle for each case and determined that while the inlet demonstrated the highest correlation coefficient, the number of abnormal CSAs were correlated with NCS severity (r=0.41, p<0.001) [30]. Singla and colleagues also found a significant correlation between CSA measurements and NCS severity, with CSA cutoffs of >13 mm^2^ for moderate cases and > 16 mm^2^ for severe cases [27]. Salman and colleagues determined that the strongest relationship between US and NCS occurred with sensory latency [25]. In addition to these findings, Visser and colleagues (2008) found that combining NCS and US was not more significantly correlated with clinical evaluation than US alone (p=0.73) [31].

Clinical correlations

In addition to assessing classic clinical symptoms of CTS, eight studies used questionnaires, including the CTS-6, BCTQ, and historical-objective (Hi-Ob) scale, as diagnostic references for NCS and US findings. The Boston questionnaire was the most used tool (n=4) [14,22,29,30], followed by the CTS-6 [17,20] and Hi-Ob scale [23,27] (n=2). Fowler and colleagues used CTS-6 as a diagnostic reference and found that US was more accurate (89%) than NCS (86%) [20]. In addition, Chen and colleagues reported that while there were significant differences between US CSA measurements for positive and negative CTS-6 scores, there was no significant difference between NCS measurements for positive and negative CTS-6 scores [17]. The two studies that used the Hi-Ob scale reported contradictory findings in which one study found significant correlations between US, NCS, and Hi-Ob scores [27], while the other study found poor correlations between the Hi-Ob scale and measurements of US and NCS [23,27]. Finally, Visser and colleagues reported significant relations between clinical severity and measurements from NCS and US, even without using a standardized diagnostic reference [31].

Other correlations and considerations

Multiple studies have found relationships between the CSA of the median nerve and demographic factors, such as age, body weight, and BMI, but the evidence is contradictory. Atan and colleagues found that age had the greatest effect on m-CSA in healthy patients, and similarly, Fowler and colleagues found that age was significantly increased in NCS-positive and US-positive patients compared with healthy subjects [20,29]. Two other studies that reported no relationship between CSAs and age provided contradictory evidence [22,30]. Two studies observed correlations between m-CSAs and increasing body weight and BMI [29,31]. One study found no correlation between m-CSAs and increasing body weight [22] while three studies found no correlation between m-CSAs and increasing BMI [22,30,31]. Visser and colleagues, the only researchers in our review to address patient preference, found that of 20 randomly selected patients, 10 had a strong or very strong preference for US compared with NCS [31]. 

Special cases

Two studies analyzed special cases of CTS, including acute CTS and atypical CTS. Due to the atypical presentation among these patients, all patients underwent both NCS and high-resolution US (HRUS). Among the acute CTS patients, the most common presenting complaint was acute onset of paresthesia of the fingers, and 44% of these patients denied an inciting event [26]. While all hands showed evidence of focal demyelination on NCS and CSA enlargement on US, 48% of patients showed persistent median artery (PMA), and 66% of those with a PMA showed a bifid median nerve [26]. Anatomic abnormalities such as PMA and bifid median nerve are important findings for perioperative planning and can only be diagnosed with US [26].

Another special case evaluated was atypical CTS, defined as classic CTS symptoms predominantly in the non-dominant hand [18]. Approximately one-third of patients (33.5%) with atypical CTS showed abnormal findings on HRUS, such as PMA, bifid or trifid median nerve, or tenosynovitis of flexor tendons, that could be causative of CTS symptoms or change the therapeutic approach [18]. The rate of structural abnormalities was significantly more frequent among patients with atypical CTS compared with typical CTS (p=0.003) [18].

Comparative sensitivity and specificity

Eleven studies provide a comparative experimental design to determine the diagnostic capabilities of NCS and US, as summarized in Table 2. US sensitivity reportedly ranged from 64.7% to 98.2%. The lower end was observed by Mondelli and colleagues, while ranges of close to 100% were observed by Singla and colleagues [27,30]. The comparison showed a higher average sensitivity for US compared with NCS (84.9% vs. 83.3%, respectively). As such, the lower sensitivity of NCS corresponded with an average false-negative rate of 24.8% based on the 11 articles. In comparison, US corresponded with an average false-negative rate of 12.5%.

**Table 2:** Highest sensitivity and specificity reported for NCS and US *Based on motor latency difference †Measured at seventh point, 3 cm distal to the distal wrist crease ‡Measured at second point, 2 cm proximal to the distal wrist crease §Based on the palm wrist distal sensory latency difference NCS, Nerve conduction studies; US, Ultrasound

Author (year) (citation)	Sensitivity US (%)	Sensitivity NCS (%)	Specificity US (%)	Specificity NCS (%)
Chen (2023) [17]	75.9	87.0	51.1	27.0
Singla (2020) [27]	98.21	82.14*	74.00	85.71*
Azadeh (2017) [16]	100^†^	96.7^†^	84.4^†^	65.2^‡^
Salman (2018) [25]	96.9	-	93.6	-
Atan (2018) [29]	79.5	-	78.5	-
Kanikannan (2015) [21]	76.43	90.53^§^	72.72	96.12^§^
Fowler (2014) [20]	89	89	90	80
Paliwal (2014) [24]	88.19	-	82.86	-
Mhoon (2012) [23]	99.0	76.0	22.0	29.0
Mondelli (2008) [30]	64.7	67.0	-	-
Kwon (2008) [22]	66.0	78.0	63.0	83.0
Mean values (%)	84.9	83.3	71.2	66.6

Similarly, US had a higher average specificity than NCS (71.2% vs. 66.6%, respectively). Kanikannan and colleagues found that NCS showed a higher specificity at 96.1% [21], while Chen and colleagues found a lower bound of specificity at 27% [16,17]. Comparatively, US specificity was highest at 93.6% and lowest at 22.0% [23,25]. As a result of its lower specificity, US has a higher average rate of false positives (30.75%) than NCS (16.46%) when diagnosing CTS. While US showed a higher average sensitivity and specificity than NCS, these differences were not statistically significant (p=0.76 and p=0.72, respectively). 

Discussion

Diagnosing CTS is a multifaceted process that includes integrating clinical presentation, diagnostic results from NCS and US, and grading severity through various peer-reviewed scales. Historically, NCS is the gold standard for diagnosing CTS and determining severity; however, the findings presented in the literature review demonstrate the increasing utility of US as a first-line diagnostic tool.

An in-depth literature analysis provided insight into the comparability of the diagnostic capabilities between US and NCS, specifically regarding sensitivity, specificity, and false-negative rate. As demonstrated in CTS studies, US showed a higher sensitivity in detecting musculoskeletal abnormalities and more robust applications for visualizing structural changes than NCS. According to Singla and colleagues, US provides clear images of nerve compression and structure, which helps in diagnosing CTS and peripheral neuropathy [27]. However, instrument quality and operator experience pose limitations [21].

Ultimately, NCS provides detailed functional information, but its sensitivity can be significantly lower when detecting early or mild abnormalities. As a result, the false-negative rate of EMG can be higher than US, especially in cases of minimal damage or when testing unaffected muscles [18].

While some variation exists across diagnostic centers regarding cut-off values for NCS in the diagnosis of CTS, there are evidence-based parameters that guide diagnostic criteria, such as the AANEM guidelines. Similar guidelines exist for US, but there is no universally accepted cut-off value for CSA of the median nerve to diagnose CTS. In this review, cut-off values ranged from 9 to 13 mm^2^, but the greatest sensitivity (100%) was recorded by using the inching technique at 3 cm distal from the distal wrist crease and a calculated cutoff of 10.98 mm^2^, which is not consistent with the current AANEM guidelines, suggesting 9-10 mm^2^ is the optimal range for accurate diagnosis [35]. While not diagnostic alone, many studies report that a WFR >1.5 in addition to an elevated CSA suggests a CTS diagnosis. In addition to these widely accepted measurements, novel measurements such as m-CSA at the outlet and TCL thickness have demonstrated clinical utility in diagnosing CTS. Measuring the m-CSA at the inlet and outlet increased the sensitivity of diagnosis from 65% to 84% in one study, while the TCL thickness showed increased correlation with the clinical severity.

While studies using standard techniques for NCS revealed sensitivities of 67% to 89%, the inching technique has shown increasing diagnostic accuracy for NCS with sensitivities of 71.87% and 96.7% at 3 cm distal to the distal wrist crease by antidromic and orthodromic analysis, respectively. While inching studies varied in the number of points measured, the 11-point inching technique did not increase the diagnostic accuracy, as all significant points were found between 3 cm distal and 2 cm proximal to the distal wrist crease for both NCS and US. These studies demonstrate that using the inching technique for US may provide more information along the length of the median nerve, but no comparisons were made between the diagnostic accuracy of the inching and the standard technique.

One advantage of US demonstrated in this review is the increased correlation between US and clinical evaluation through diagnostic questionnaires. Two studies found that CTS-6 scores were more strongly associated with US findings than NCS measurements. One study stated that US was more accurate than NCS regarding CTS-6 scores, and the other study found significant differences between US measurements of positive and negative CTS-6 scores, with no significant differences observed for NCS. Studies using the Hi-Ob scale were contradictory, with one describing significant correlations between the Hi-Ob scale, US, and NCS, and the other reporting no significant correlations between the Hi-Ob scale and US or NCS findings.

One often-discussed disadvantage of using US to diagnose CTS is that it cannot determine disease severity as NCS can, with increasing latencies. This review shows a strong correlation between increasing m-CSA and severity measured by the Stevens and Padua scales, the most cited among studies in this review. One study delineated that CSA cutoffs of >13 mm2 coincided with moderate cases and >16 mm2 coincided with severe cases [27]. The limitation of this comparison is that some studies defined cases of CTS by NCS findings rather than clinical presentation, but this difference did not determine which studies found a correlation. This limitation, however, demonstrates the need for further research investigating US findings in clinical CTS without NCS confirmation.

The limitations of this study include its design, the availability of quality studies for analysis, and conflicting evidence among studies. Although this work represents a comprehensive literature review with robust methodology and detailed analyses, the level of evidence produced by a systematic review with meta-analysis is inherently stronger. Furthermore, the specific focus of this research limited both the number and quality of studies eligible for inclusion. Finally, many of the included studies demonstrated conflicting findings, suggesting heterogeneity in study populations, methodologies, accuracy measures, and overall quality of evidence, thereby highlighting the need for further high-quality research.

Future directions

Based on the findings of this literature review, significant gaps exist regarding the clinical utility of NCS and US for diagnosing CTS. First, many studies defined cases of CTS based on findings from NCS. This strategy could exclude cases that may only be diagnosed through the US; however, including CTS cases defined by NCS did not determine which studies found a correlation between severity and US findings. Second, only one study included parameters measuring patient preference, finding that US was preferable to patients undergoing CTS diagnosis. Third, only one study discussed the advantages of using US to guide surgical planning but provided no objective measures regarding preference or benefit from surgeons. Finally, only two studies analyzed the diagnostic utility of the inching technique, but there was no comparison to the standard technique for either NCS or US. Future research should explore the efficacy of US as a standalone diagnostic tool, examine patient preferences, assess the benefits of surgical planning, and evaluate the enhanced diagnostic capabilities of inching techniques compared to standard NCS and US evaluations.

## Conclusions

In this review of CTS diagnostic methods, we found that NCS remains the traditional standard of care but has been increasingly complemented by US due to its high sensitivity, ease of use, and patient preference. US demonstrates high sensitivity in visualizing musculoskeletal structures and identifying anatomical abnormalities such as persistent median arteries and bifid nerves, which are valuable for surgical planning. At the same time, NCS provides detailed functional insights, which are especially useful for determining severity based on latency measures. Although NCS is considered superior in determining the severity of CTS, this review shows that the US has demonstrated increasing correlations between m-CSA and the clinical severity of CTS. Novel US measurements such as the transverse carpal ligament thickness and median nerve cross-sectional area at the carpal tunnel outlet add potential diagnostic value, but US lacks universal severity scales similar to NCS. Despite the advantages of US, gaps in research persist, such as its standalone diagnostic capability without NCS confirmation, its role in surgical planning, and comprehensive patient preference studies. Future studies should focus on refining these areas to optimize CTS diagnostic accuracy and patient care.
